# High-voltage impedance rise; mechanism and management in patients with transvenous implantable cardioverter-defibrillators: a case series

**DOI:** 10.1093/ehjcr/ytz220

**Published:** 2019-12-19

**Authors:** Christopher Monkhouse, Alex Cambridge, Anthony W C Chow, Jonathan M Behar

**Affiliations:** Department of Cardiac Electrophysiology, Barts Heart Centre, West Smithfields, London EC1A 7BE, UK

**Keywords:** Shock impedance, Lead failure, ICD, Implantable cardioverter-defibrillator, Encapsulation, Case report

## Abstract

**Background:**

We describe a case series of patients for a gradual rise in daily, low-voltage sub-threshold measurement (LVSM) of shock (high-voltage, HV) impedance in a group of patients with Boston Scientific implantable cardioverter-defibrillators (ICDs) and investigate the cause of the abnormality.

**Case summary:**

Six patients presented with a gradual rise in HV impedance above normal range (132.5 ± 20.8 Ω). Patients were young with a mean age of 29 ± 11 years, four patients had hypertrophic cardiomyopathy, one left ventricular non-compaction, and one long QT. All lead designs were silicon body with GORE polytetrafluoroethylene (ePTFE) coated coils, and a lower true shock impedance (TSI) was seen in all cases with full output synchronized shock. We compared the rate of HV impedance rise with our historical cohort of Boston ICDs using an unpaired *t*-test. The change in impedance per month was significantly higher amongst our six patients when compared with our cohort of Boston Scientific ICDs (3.2 ± 1.9 Ω/month vs. 0.0008 ± 0.005 Ω/month, *P* < 0.001). Patients were individually investigated and management discussed in a dedicated device multi-disciplinary team meeting (MDT).

**Discussion:**

There are distinct differences between TSI and LVSM. The TSI is derived from a full output shock, whilst LVSM is calculated from a small current output. These cases highlight the inaccuracies of the LVSM measurement. The gradual rise in LVSM is significantly higher than the value for TSI in these patients we propose the most likely mechanism is encapsulation fibrosis surrounding the right ventricular shock coil. Management for these patients requires vigorous testing to rule out electrical failure, and replacement maybe necessary.

**Learning points**

- Gradual rise in implantable cardioverter-defibrillator shock impedance may be caused by encapsulation fibrosis; this may be seen in isolation and in the absence of electrical failure.
- Synchronized shock through the device can be used to determine whether a true lead integrity issue exists and requires replacement.
- Low-voltage subthreshold measurements may be deceptive and small changes to true impedance can be amplified. Only a true shock impedance can give an accurate impedance reading.

## Introduction

Effective defibrillation requires sufficient current across the myocardial mass to reset the action potentials of a critical number of myocardial cells. An implantable cardioverter-defibrillator (ICD) system with a high impedance will have greater resistance to current flow and defibrillation may be unsuccessful. Ensuring ongoing integrity of the high-voltage (HV) system is an essential part of the device monitoring process. Defibrillation impedance (also termed shock or HV impedance) can be measured in two different ways: either through a high output therapy—termed true shock impedance (TSI); or through a calculated value derived using a low voltage sub-threshold measurement (LVSM).^2–4^ True shock impedance is rarely performed as a routine test as it requires sedating the patient to deliver a shock through the device. The normal range for HV impedance is approximately 30–110 Ω from a single coil circuit and 20–70 Ω in a dual coil circuit, with a small variance between lead designs.^5^

Acute impedance rises can occur post-implantation due to incomplete pin engagement or from a pneumothorax and gradual changes in HV impedance up to 12 Ω have been described in the first 3 months of follow up.^6^ This could be due to lead movement or because of initial encapsulation of the right ventricular (RV) coil, which reduces its contact with the blood pool, increasing the coil shock impedance. There is limited evidence on gradual changes in impedance beyond 1 year.^6^^,^^7^ Causes for this have been attributed to metal ion oxidization (MIO) and environmental stress cracking (ESC).^5^ Metal ion oxidization is a degradation process caused by exposure of polyurethane to metal ions released by the conductor elements. This can be due to chemical oxidization, solvation, galvanic, or electrolytic corrosion. These ions cause cracks that expose the metal conductor elements to body fluids creating a chain reaction and gradually increasing impedance.^5^ Environmental stress cracking is caused by shocking coil oxidation which weakens the surface structure causing brittle microcracks. These cracks are then exposed to further chemical reactions that cause a gradual rise in impedance.

Here, we present a case series of patients implanted with Boston Scientific ICDs, all of whom exhibited a significant, but gradual, rise in HV impedance above the upper limit of normality. We compared these cases to our historical ICD dataset of similar devices from the same manufacturer to identify any differences.

## Timeline

**Table:** 

Patient 1	
Pre-implantation	Hypertrophic cardiomyopathy, primary prevention dual chamber implantable cardioverter-defibrillator (ICD)
Implant 2007	A dual coil right ventricular (RV) lead was implanted
	The high-voltage (HV) impedance (low-voltage sub-threshold measurement, LVSM) at implant was 58 Ω in dual coil configuration
February 2017	The HV impedance (LVSM) begins to rise
June 2017	The HV impedance (LVSM) had risen to 167 Ω in dual coil configuration
August 2017	A full output synchronized shock was performed showing a true shock impedance (TSI) of 116 Ω in dual coil configuration
May 2018	Right ventricular lead was replaced at time of upgrade to cardiac resynchronization defibrillator (CRT-D)
	New implant HV impedance (LVSM) of 53 Ω
Patient 2	
Pre-implantation	Hypertrophic cardiomyopathy, primary prevention dual chamber ICD
Implant 2012	A single coil RV lead was implanted
	The HV impedance (LVSM) at implant was 50 Ω
June 2016	The HV impedance (LVSM) begins to rise
January 2018	The HV impedance (LVSM) had risen to 118 Ω
February 2018	A full output synchronized shock was performed showing a TSI of 76 Ω in dual coil configuration
	Continued monitoring of RV lead as TSI normal
Patient 3	
Pre-implantation	Hypertrophic cardiomyopathy, primary prevention dual chamber ICD
Implant 2006	A dual coil RV lead was implanted
	The HV impedance (LVSM) at implant was 38 Ω in dual coil configuration
October 2015	The HV impedance (LVSM) begins to rise
April 2018	The HV impedance (LVSM) had risen to 126 Ω in dual coil configuration
June 2018	A full output synchronized shock was performed showing a TSI of 78 Ω in dual coil configuration
	Continued monitoring of RV lead as TSI normal
Patient 4	
Pre-implantation	Long QT syndrome, secondary prevention dual chamber ICD
Implant 2013	A single coil RV lead was implanted
	The HV impedance (LVSM) at implant was 55 Ω
May 2016	The HV impedance (LVSM) begins to rise
January 2017	The HV impedance (LVSM) had risen to 125 Ω
February 2017	A full output synchronized shock was performed showing a TSI of 90 Ω
August 2017	Right ventricular lead extraction was elected after discussion with guardians
	Extraction unsuccessful, unable to retrieve RV coil and tip due to fibrous adhesions with a mechanical sheath. New HV impedance (LVSM) of 54 Ω
Patient 5	
Pre-implantation	Hypertrophic cardiomyopathy, secondary prevention dual chamber ICD
Implant 2003	A dual coil RV lead was implanted
	The HV impedance (LVSM) at implant was 55 Ω in dual coil configuration
December 2015	The HV impedance (LVSM) begins to rise
March 2018	The HV impedance (LVSM) had risen to 107 Ω in dual coil configuration and 135 Ω in single coil configuration
April 2018	A full output synchronized shock was performed showing a TSI of 76 Ω in single coil configuration
	Continued monitoring of RV lead as TSI normal
Patient 6	
Pre-implantation	Left ventricular non-compaction, primary prevention dual chamber ICD
Implant 2005	A dual coil RV lead was implanted
	The HV impedance (LVSM) at implant was 56 Ω in dual coil configuration
February 2008	The HV impedance (LVSM) begins to rise
February 2018	The HV impedance (LVSM) had risen to 137 Ω in dual coil configuration and 154 Ω in single coil configuration
March 2018	A full output synchronized shock was performed showing a TSI of 121 Ω in single coil configuration
	A new RV lead was added at time of box change as patient declined extraction

## Results

**Figure 1:**
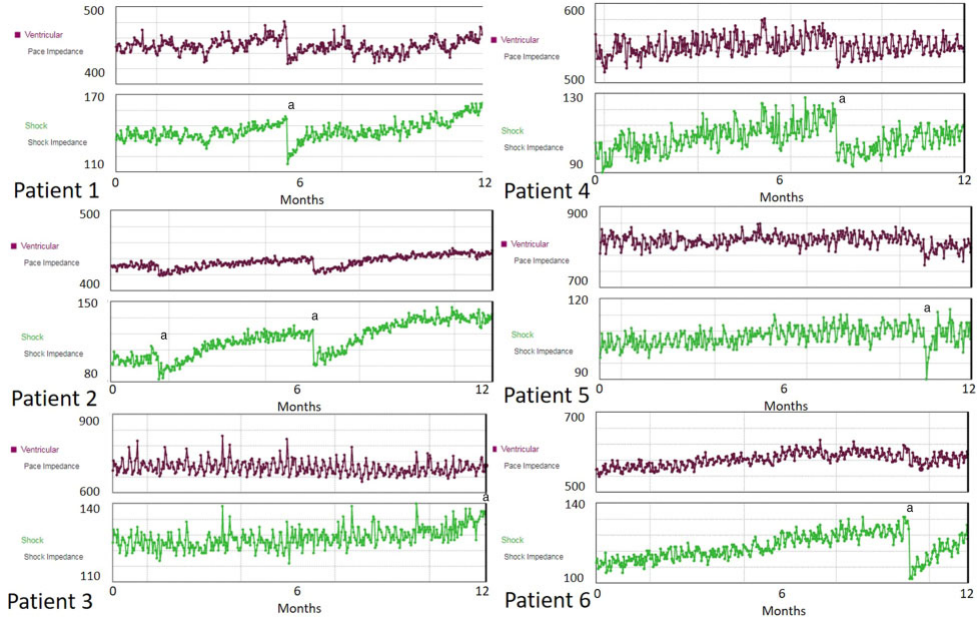
Shock impedance and bipolar right ventricular impedance (Ω) trends for each patient over the past year. ^a^Highlights the when a high-voltage therapy was performed.

**Figure 2:**
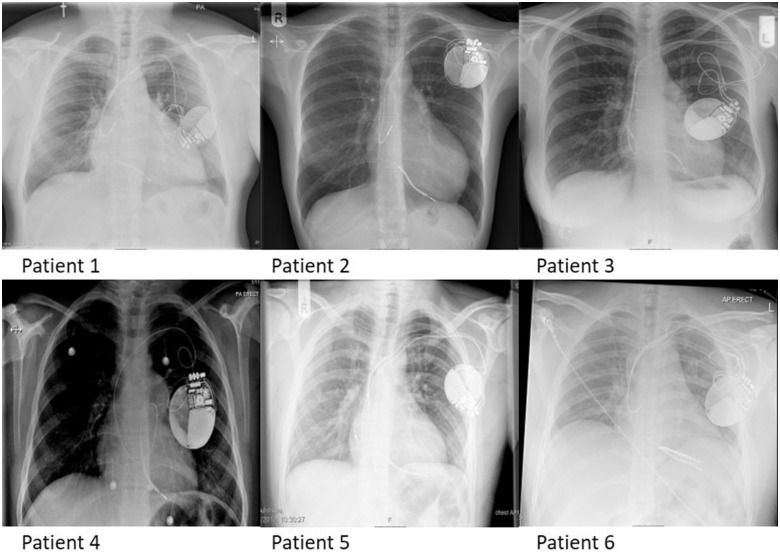
Anteroposterior chest radiographs performed for each patient.

This case series describes six patients (*Table 1*) with low-voltage sub-threshold measurement (LVSM) values significantly higher than at initial implant (*Figure 1*). After reviewing each case in turn, the impedance rise did not correlate with any specific clinical event such as decompensated heart failure, pulmonary oedema, pneumothorax, or trauma, nor as a result of any change in medication. Furthermore, blood tests confirmed no electrolyte abnormalities in any of our patients. All leads had Gore polytetrafluoroethylene (ePTFE) coated coils. Chest radiographs showed no macroscopic appearances of a lead insulation breaches (*Figure 2*) and for the three patients that had procedures, there was no evidence of pocket calcification. Echocardiography was unable to demonstrate any evidence of exaggerated lead fibrosis for all cases. Patient 4 was the first patient presented in this series. Following an open discussion with the patient and their parents, a decision for attempted extraction and reimplantation was made.

**Table 1:** Summary of patient details with shock impedance values measured in DC or SC

Patient ID	Age	Aetiology	ECG indication	Device position	Implant	Lead details	Coils	Access	LVSM at implant (Ω)	Rate of rise in LVSM (Ω/month)	LVSM prior to shock (Ω)	TSI (Ω)	Management
1	43	HCM	Primary prevention, LBBB	Sub-pec	01 March 2007	Guidant 0185	Dual coil	Subclavian	58 (DC)	6 (DC)	167 (DC)	116 (DC)	New RV lead added at time of upgrade to CRT. New LVSM 53 Ω
2	22	HCM	Primary prevention, normal QRS	Sub-pec	20 December 2012	Boston Scientific 0292	Single coil	Axillary	50 (SC)	4.24 (SC)	118 (SC)	76 (SC)	Continued monitoring
3	38	HCM	Primary prevention, LBBB	Sub-pec	18 July 2006	Guidant 0165	Dual coil	Cephalic	38 (DC)	2.93 (DC)	126 (DC)	78 (DC)	Continued monitoring
4	12	Long QT	Secondary prevention, normal QRS	Sub-pec	February 2013	Boston Scientific 0692	Single coil	Axillary	55 (SC)	4.25 (SC)	140 (SC)	90 (SC)	Attempted RV lead extraction and lead replacement, unable to retrieve RV coil. New LVSM 54 Ω
5	27	HCM	Secondary prevention, RBBB	Sub-pec	16 October 2003	Guidant 0165	Dual Coil	Cephalic	55 (DC)	1.01 (DC)	107 (DC) 135 (SC)	76 (SC)	Continued monitoring
6	32	Non- compaction	Primary prevention, normal QRS	Sub-cut	01 September 2005	Guidant 0165	Dual coil	Cephalic	56 (DC)	0.51 (DC)	137 (DC) 154 (SC)	121 (SC)	New RV lead added at time of box change. New LVSM 52 Ω

DC, dual coil; SC, single coil.

### Mean change in impedance amongst implantable cardioverter-defibrillators

We used our historical data from patients previously implanted with Boston Scientific ICDs (n1,477) using the Latitude system to document the mean change in shock impedance over the course of a 12-month period. Any patients with suspected shock conductor fractures were excluded from this calculation. Using an unpaired *t*-test, the change in impedance per month was significantly higher amongst our case series of patients compared with the cohort with Boston Scientific ICDs (3.2 ± 1.9 Ω/month vs. 0.0008 ± 0.005 Ω/month, *P* < 0.001).

## Discussion

### True shock impedance vs. low-voltage subthreshold impedance measurements

There are distinct differences between a TSI and a LVSM. Device-based diagnostics now place greater demands on battery current and many manufacturers have reduced the energy used to obtain the LVSM. Since the Teligen generation of Boston Scientific ICDs, Boston Scientific used a smaller electrical impulse of 80 µA@ 156 µs to measure the LVSM. This impulse was less than previous models which used 15 mA @ 60 µs.^8^ Of note, Abbott and Biotronik devices also use small current impulses (750 µA@ 19 ms, 1 mA @ 30 µs, respectively). These smaller current calculated values can be affected by subtle changes to current flow, causing minor variation in impedance values day to day. These are caused by external and internal factors such as; positional, external electromagnetic interference, and electrolyte balance. Medtronic ICDs use a higher sub-threshold constant voltage stimulus (1 V @90 µs) coupled with mathematical scaling to arrive at the projected HV therapy pulse, making it less variable. In our patients, whilst the shock impedance trends show daily fluctuations (due to causes described above), the ongoing rising trend in impedance suggests an additional contributing factor (*Figure 1*).

### What is the mechanism of rising high-voltage impedance?

Patients assessed in device clinic where provocative manoeuvres (arm manipulation, pectoral muscle contraction, pocket manipulation, deep expiration, and inspiration both standing and supine) were performed showed no signs of lead malfunction or baseline wander on right ventricle and far field electrograms. For dual coil leads, individual vector breakdown showed stable SVC coil measurements and abnormally high RV coil measurements. Furthermore, in all cases, the rise in HV impedance correlated with a Superior Vena Cava (SVC), gradual rise in RV pacing impedance over time. This is logical as all six leads have integrated bipolar design, which measures the RV pacing impedance between the RV tip and RV coil. This isolates the problem to the RV coil.

Two mechanisms have previously been described for gradual rises in shock impedance, MIO, and ESC. These mechanisms, however, do not fit this presentation, because the TSI should be an abnormally high measurement, not a normalised one, as the coil conductor is damaged. One explanation for our findings could be encapsulation of the RV coil by endocardium and an additional tissue fibrotic process. Tissue growth around endocardial defibrillator leads has been widely reported in the literature, from extraction studies, post-transplant and post-mortem examination. It is likely to be the cause of the rise in LVSM post-implant.^5^^,^^10–16^ However, proving that there is a definitive fibrotic or calcification process in these patients is difficult without surgical lead extraction. Imaging modalities such as echocardiography, cardiac magnetic resonance imaging and cardiac computed tomography suffer from artefact as a consequence of the metal lead conductors.

Encapsulation may cause a falsely high LVSM; due to rising tissue density surrounding the coil. However, the TSI would be less affected as the value is not scaled, producing a reduced, normalised value. This would also explain why this phenomenon is only being seen in newer generation devices that have smaller LVSM energy outputs. Furthermore, four relatively young patients in this series have a diagnosis of hypertrophic cardiomyopathy; one may postulate that a more aggressive fibrotic reaction in these patients may exacerbate the fibrotic process contributing to endocardial encapsulation.^17^

One commonality between all patients is that they all had GORE ePTFE coated coils. When GORE ePTFE coated coils were initially produced there was a warning that one may expose the coil by damaging the coating if not implanted correctly. This could prove an additional theory as it may result in infiltration of the exposed conductor and a rise in impedance.^18^ However, we observed no electrical abnormality and an exposed coil should produce an abnormal TSI.

Another theory is ionic build up around the RV coil. This may be caused by the anodal shock vector attracting free floating electrons to the RV coil, causing a gradual rise in shock impedance. An HV shock would dissipate these ions, producing a value comparable to implant. However, in our series, all values of TSI have been raised compared to implant suggesting that there is an underlying issue surrounding the RV coil. Also, all patients had normal blood electrolyte values which would make this theory less likely to be the sole cause. Ionic build up that is dissipated with synchronised shock may prove a reason as to why the LVSM transiently drops post-shock before increasing once more.

From our case series, we have only observed this phenomenon in Boston Scientific devices and leads. Whether this is a unique problem applicable to Boston leads remains uncertain as this manufacturer makes up the largest proportion of the ICDs implanted at our centre. Therefore, our findings may extend to other manufacturers with time and increased volume.

### Management of defibrillator systems with rising high-voltage impedance

**Figure 3:**
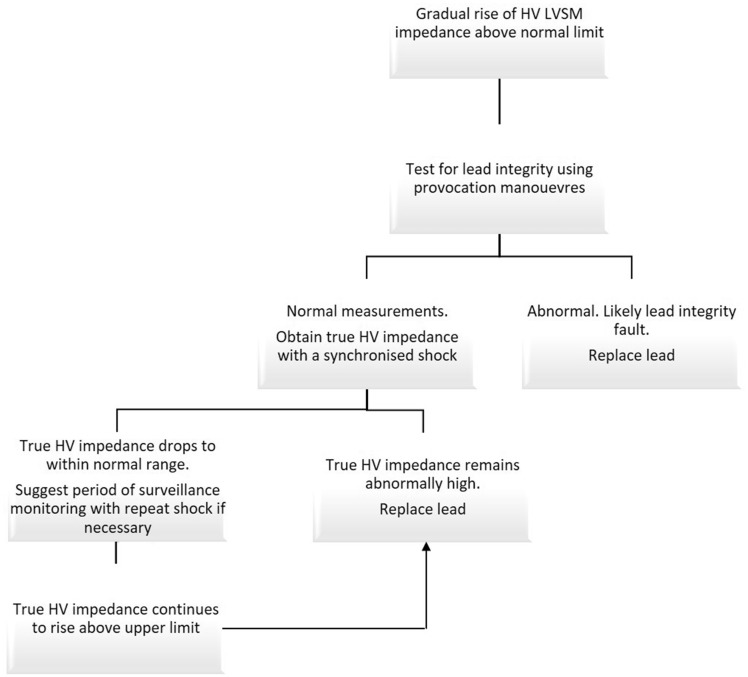
Flow diagram of suggested management for patients with high-voltage impedance measurements on Boston Scientific ICDs. HV, high voltage; LVSM, low-voltage sub-threshold measurement.

A full clinical assessment is an essential part of the investigative process. One may speculate that one could predict such an issue from the rate of increase in the impedance, however, from just six cases, there is insufficient data to make such inferences. Over the past 12 months, the average rise in LVSM per month of 3.2 ± 1.9 Ω/month was seen across the six cases. This certainly appears higher than the trends from our historical Boston ICD dataset. A suggested management flow diagram is demonstrated in *Figure 3*. Of note, if patients are to be considered for transvenous lead extraction, one may consider this a higher risk procedure, due to potentially more extensive tissue fibrosis or infiltration of the RV coil. Interestingly, we were unable to retrieve the RV coil with a mechanical sheath in Patient 4 due to extensive tethering to the intracardiac tissue. For patients that have normal TSI the active monitoring will continue with synchronised shocks needing to be performed as the LVSM is inaccurate. Whilst this does not resolve the problem, it may prevent unnecessary lead extraction and replacement.

## Conclusion

Gradual rises in LVSM may be attributed to encapsulation of RV coils as well as MIO and ESC. The phenomena can be distinguished by a normalized TSI when measured with a full output synchronised shock compared to the LVSM. Managing these leads requires vigorous testing and replacement may be necessary. For those who have normal TSI, close monitoring is required to ensure a sufficient impedance to effectively defibrillate the patient.

## Lead author biography

**Figure:**
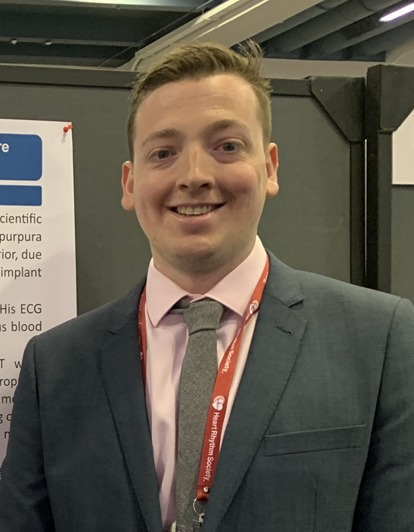


Christopher Monkhouse, BSc CCDS. Cardiac electrophysiology has been my passion since starting my training in 2011. Being a cardiac patient, myself has given me special insight into the patient’s perspective of health services, driving me to provide the highest level of evidence-based care for patients within Cardiac Rhythm Management, in particular device therapy and arrhythmia.

## Supplementary material

Supplementary material is available at *European Heart Journal - Case Reports* online.

**Slide sets:** A fully edited slide set detailing this case and suitable for local presentation is available online as Supplementary data.

**Consent:** The author/s confirm that written consent for submission and publication of this case report including image(s) and associated text has been obtained from the patient in line with COPE guidance.

**Conflict of interest:** C.M. has received speaker fees from Abbott Ltd. A.W.C. has received grant fees from Abbott Ltd. J.B. is an associate editor of EHJ-Case Reports, received speaker fees from Abbott Ltd and research grant from Boston Scientific.

## Supplementary Material

ytz220_Supplementary_Slide_Set
