# Supplementary material for: High-voltage impedance rise; mechanism and management in patients with transvenous implantable cardioverter-defibrillators: a case series
Source: Eur Heart J Case Rep. 2019 Dec 19;3(4):1–8. doi: 10.1093/ehjcr/ytz220 (PMC6939807; doi:10.1093/ehjcr/ytz220)
Supplement: ytz220_Supplementary_Slide_Set [file ytz220_supplementary_slide_set.pptx]

## Slide 1
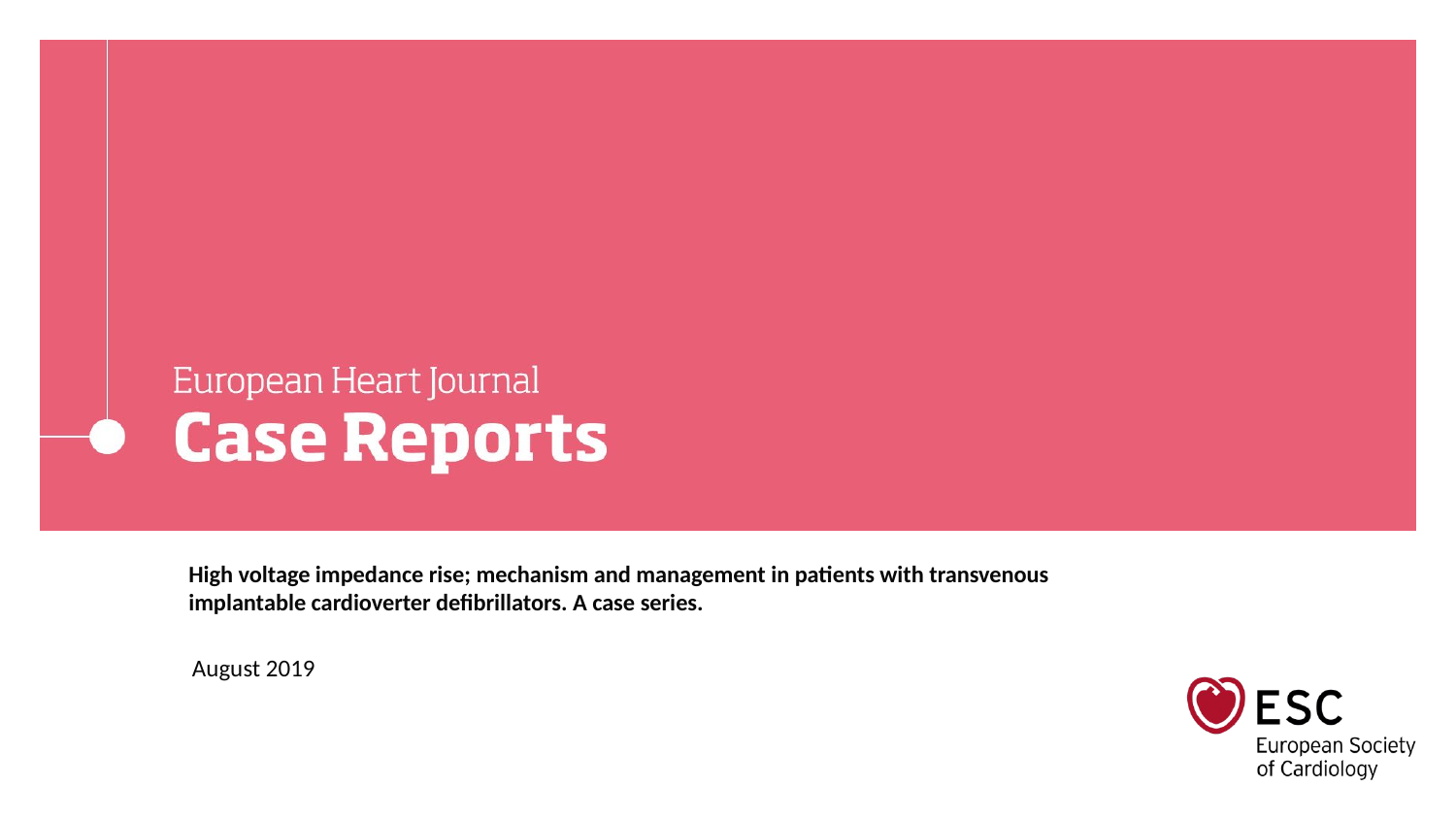

# High voltage impedance rise; mechanism and management in patients with transvenous implantable cardioverter defibrillators. A case series.
August 2019

## Slide 2
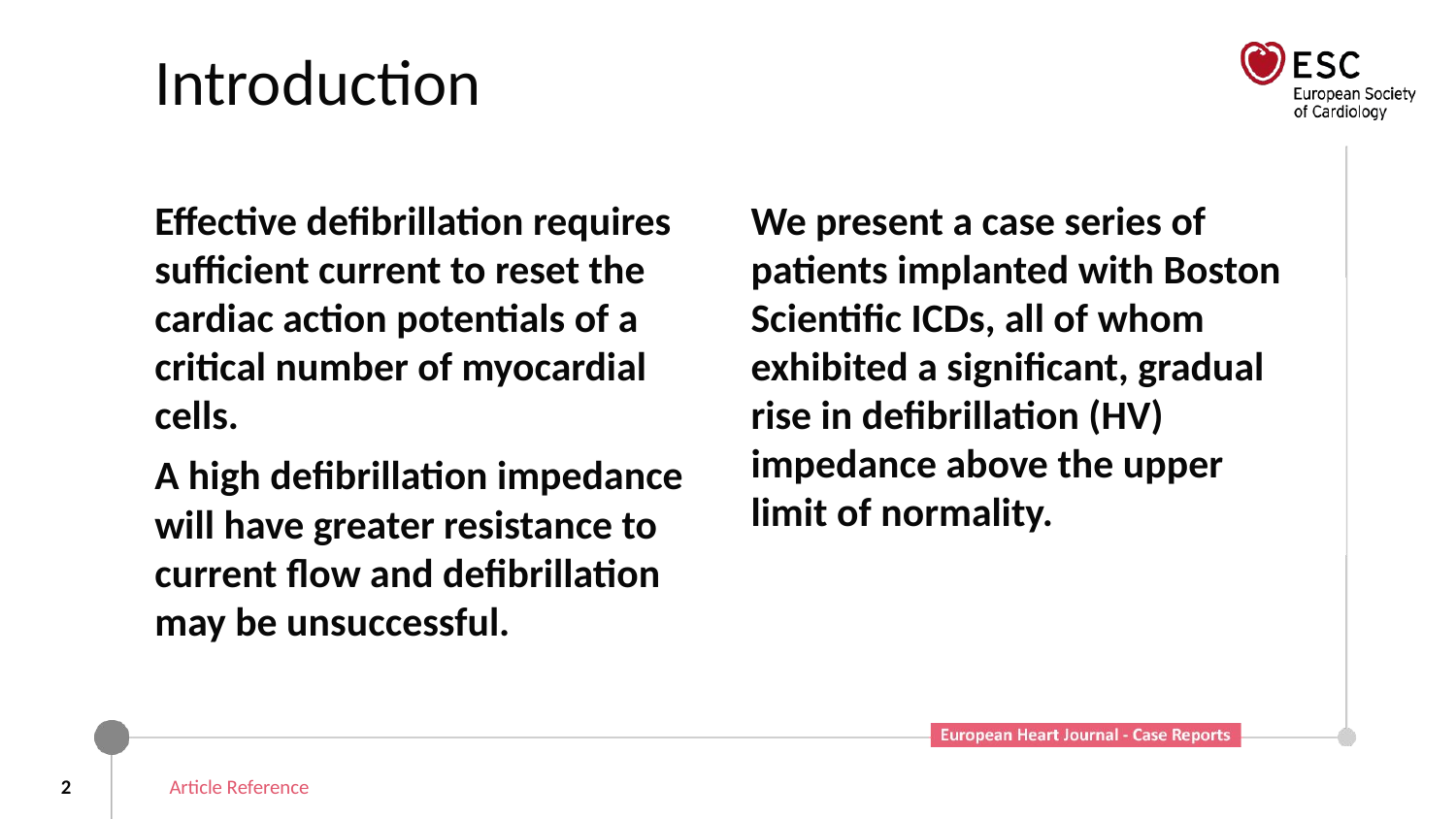

# Introduction
Effective defibrillation requires sufficient current to reset the cardiac action potentials of a critical number of myocardial cells.
A high defibrillation impedance will have greater resistance to current flow and defibrillation may be unsuccessful.
We present a case series of patients implanted with Boston Scientific ICDs, all of whom exhibited a significant, gradual rise in defibrillation (HV) impedance above the upper limit of normality.
2
Article Reference

## Slide 3
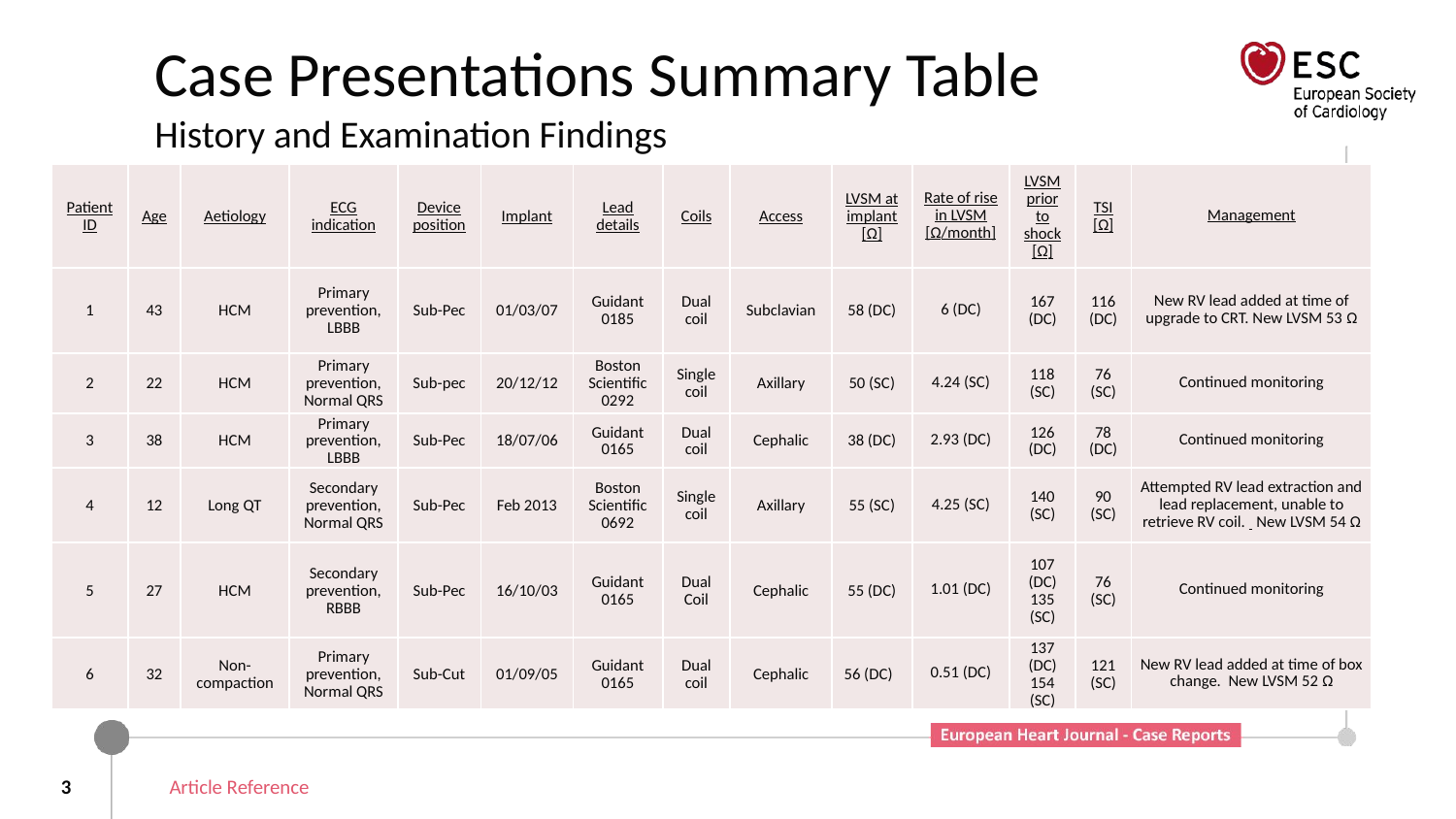

# Case Presentations Summary Table History and Examination Findings
| Patient ID | Age | Aetiology | ECG indication | Device position | Implant | Lead details | Coils | Access | LVSM at implant [Ω] | Rate of rise in LVSM [Ω/month] | LVSM prior to shock [Ω] | TSI [Ω] | Management |
| --- | --- | --- | --- | --- | --- | --- | --- | --- | --- | --- | --- | --- | --- |
| 1 | 43 | HCM | Primary prevention, LBBB | Sub-Pec | 01/03/07 | Guidant 0185 | Dual coil | Subclavian | 58 (DC) | 6 (DC) | 167 (DC) | 116 (DC) | New RV lead added at time of upgrade to CRT. New LVSM 53 Ω |
| 2 | 22 | HCM | Primary prevention, Normal QRS | Sub-pec | 20/12/12 | Boston Scientific 0292 | Single coil | Axillary | 50 (SC) | 4.24 (SC) | 118 (SC) | 76 (SC) | Continued monitoring |
| 3 | 38 | HCM | Primary prevention, LBBB | Sub-Pec | 18/07/06 | Guidant 0165 | Dual coil | Cephalic | 38 (DC) | 2.93 (DC) | 126 (DC) | 78 (DC) | Continued monitoring |
| 4 | 12 | Long QT | Secondary prevention, Normal QRS | Sub-Pec | Feb 2013 | Boston Scientific 0692 | Single coil | Axillary | 55 (SC) | 4.25 (SC) | 140 (SC) | 90 (SC) | Attempted RV lead extraction and lead replacement, unable to retrieve RV coil. New LVSM 54 Ω |
| 5 | 27 | HCM | Secondary prevention, RBBB | Sub-Pec | 16/10/03 | Guidant 0165 | Dual Coil | Cephalic | 55 (DC) | 1.01 (DC) | 107 (DC) 135 (SC) | 76 (SC) | Continued monitoring |
| 6 | 32 | Non-compaction | Primary prevention, Normal QRS | Sub-Cut | 01/09/05 | Guidant 0165 | Dual coil | Cephalic | 56 (DC) | 0.51 (DC) | 137 (DC) 154 (SC) | 121 (SC) | New RV lead added at time of box change. New LVSM 52 Ω |
3
Article Reference

## Slide 4
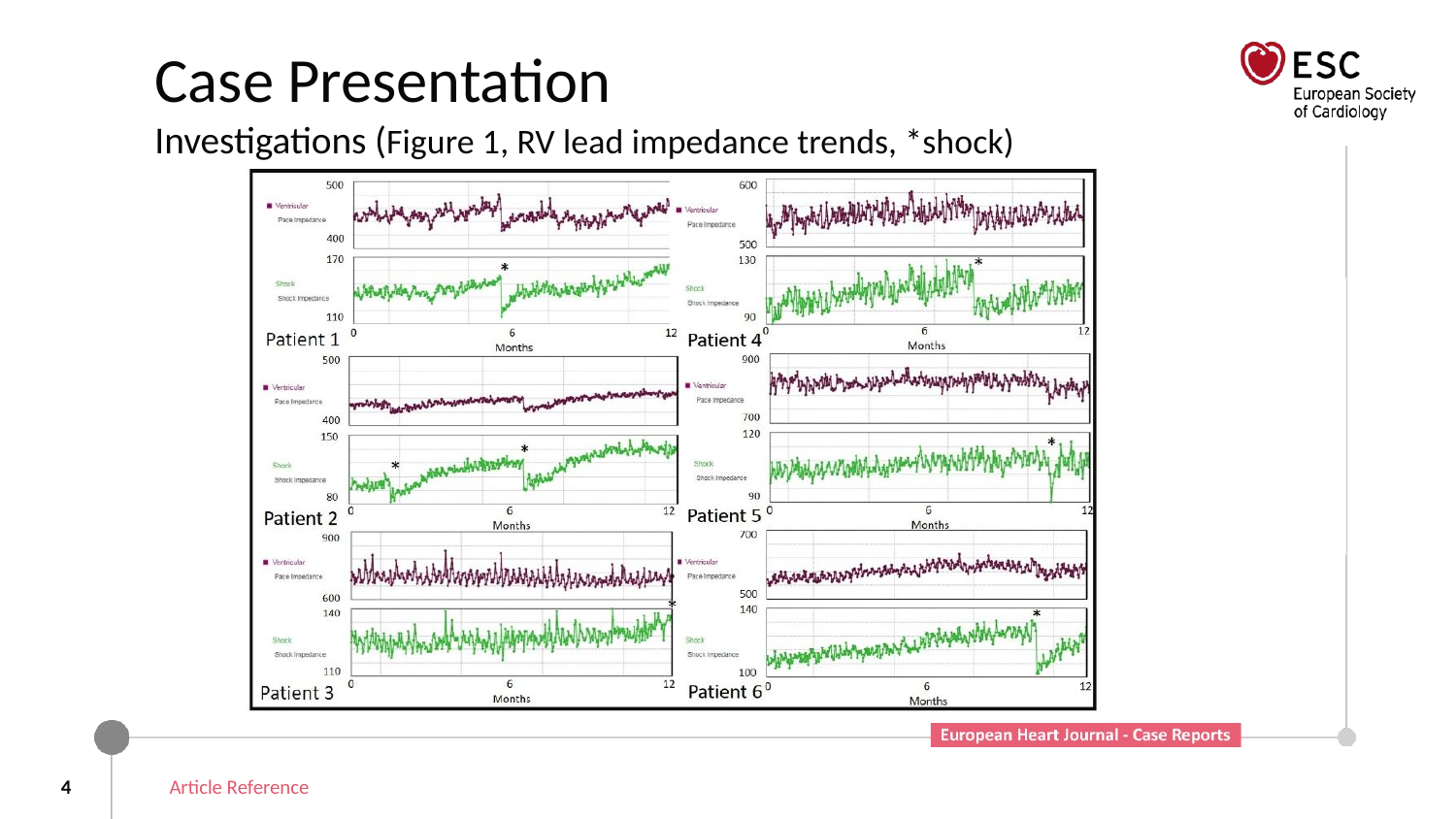

# Case Presentation Investigations (Figure 1, RV lead impedance trends, *shock)
4
Article Reference

## Slide 5
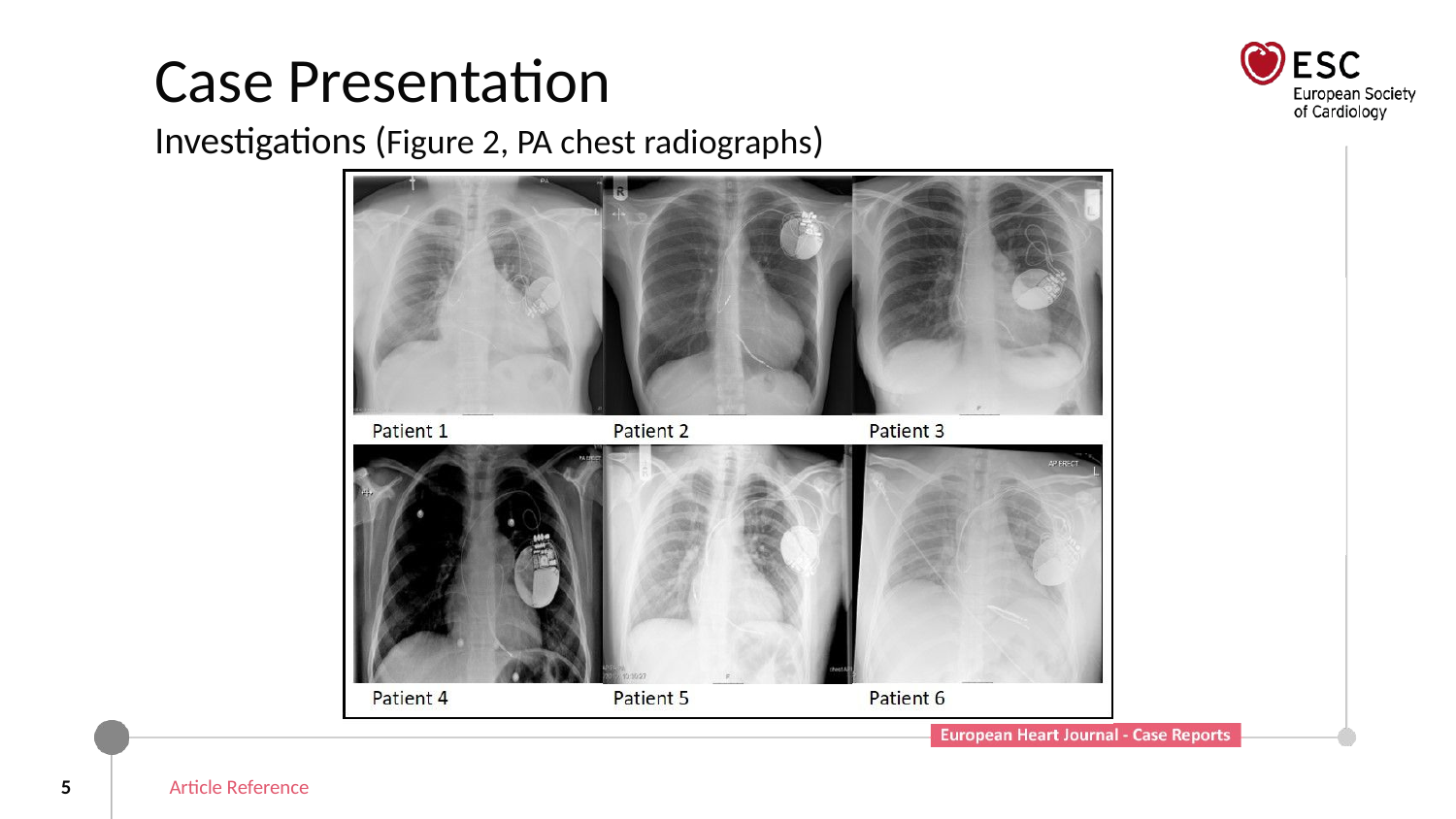

# Case Presentation Investigations (Figure 2, PA chest radiographs)
5
Article Reference

## Slide 6
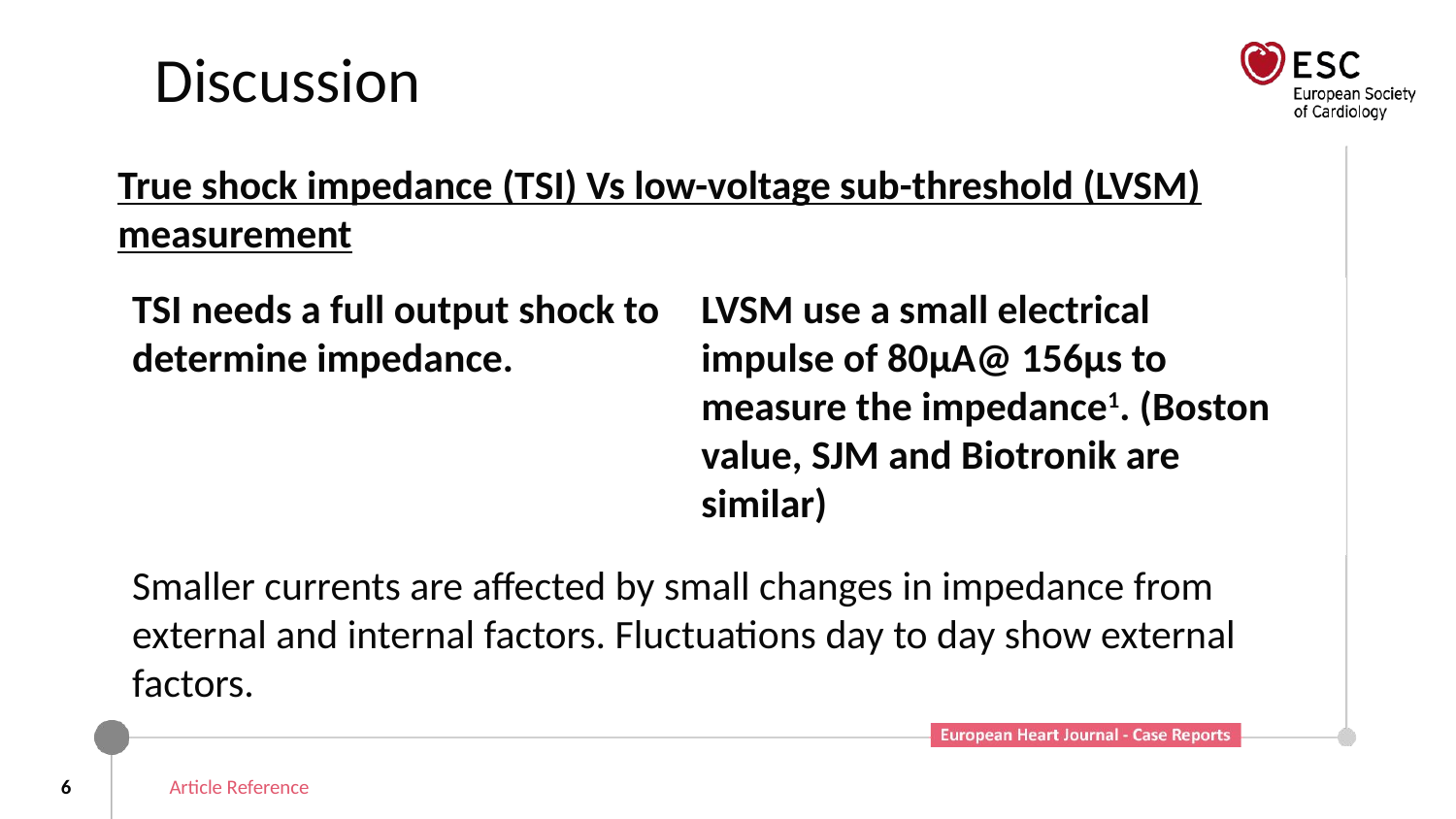

# Discussion
True shock impedance (TSI) Vs low-voltage sub-threshold (LVSM) measurement
TSI needs a full output shock to determine impedance.
LVSM use a small electrical impulse of 80µA@ 156µs to measure the impedance1. (Boston value, SJM and Biotronik are similar)
Smaller currents are affected by small changes in impedance from external and internal factors. Fluctuations day to day show external factors.
6
Article Reference

## Slide 7
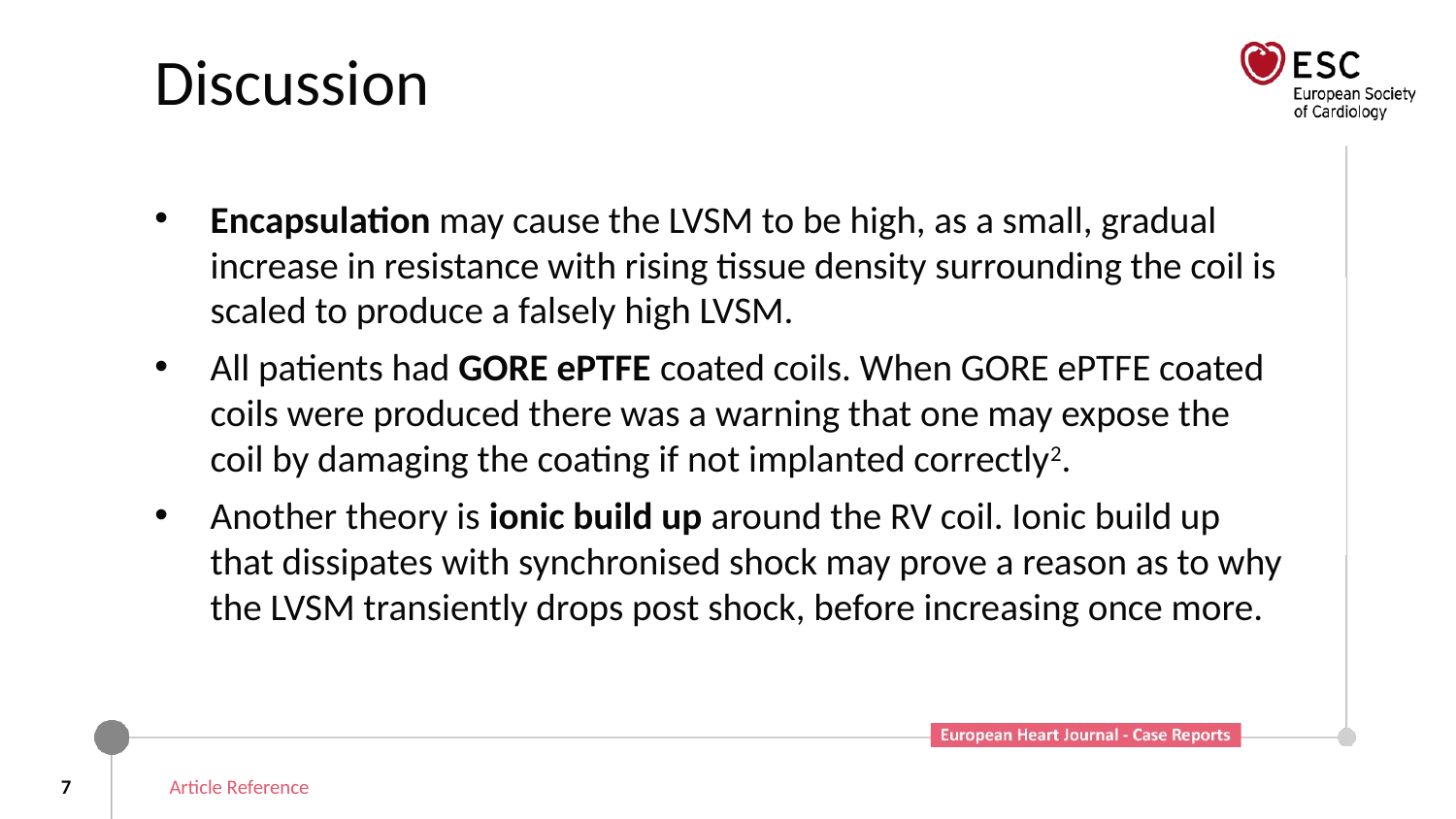

# Discussion
Encapsulation may cause the LVSM to be high, as a small, gradual increase in resistance with rising tissue density surrounding the coil is scaled to produce a falsely high LVSM.
All patients had GORE ePTFE coated coils. When GORE ePTFE coated coils were produced there was a warning that one may expose the coil by damaging the coating if not implanted correctly2.
Another theory is ionic build up around the RV coil. Ionic build up that dissipates with synchronised shock may prove a reason as to why the LVSM transiently drops post shock, before increasing once more.
7
Article Reference

## Slide 8
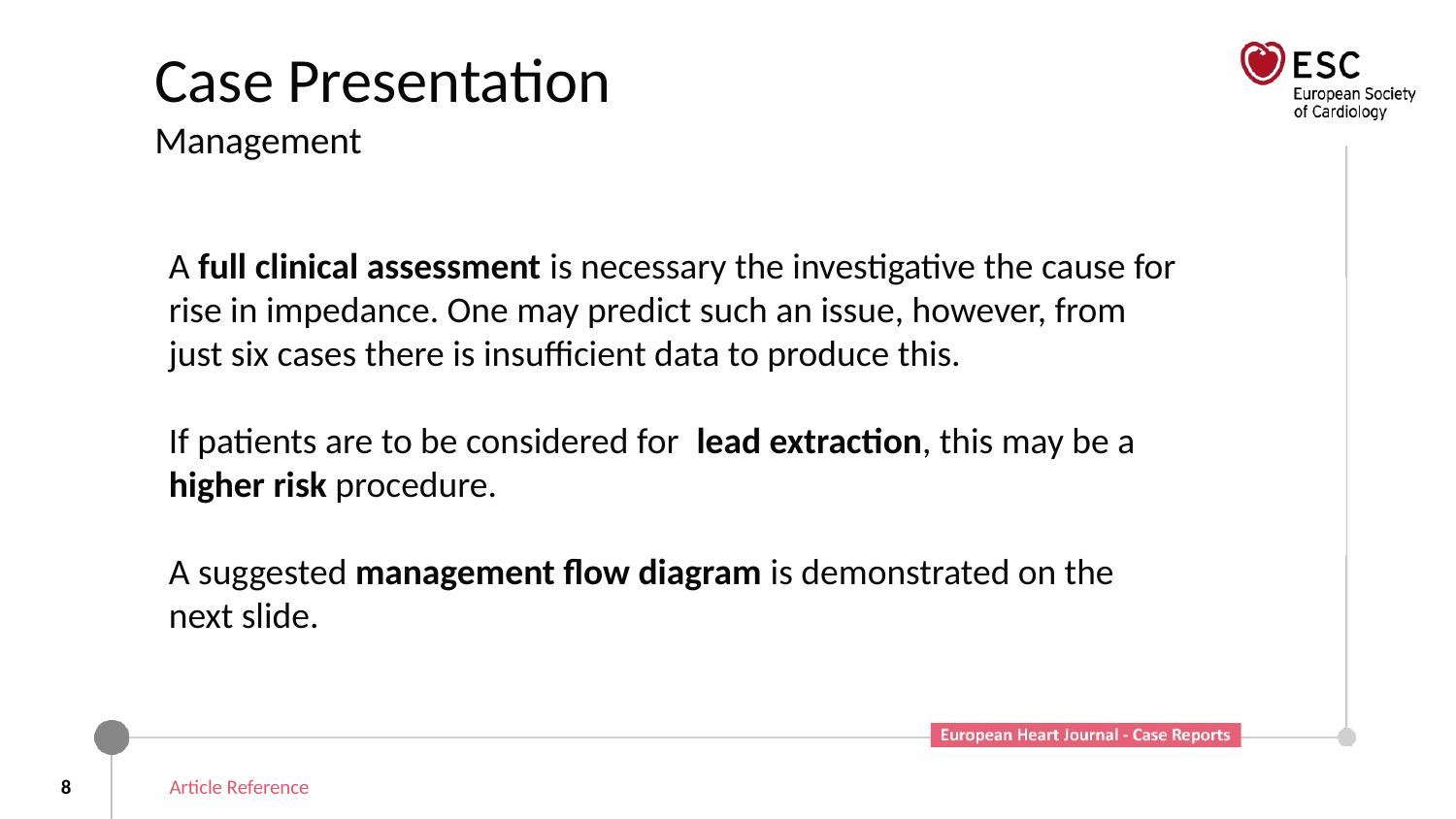

# Case Presentation Management
A full clinical assessment is necessary the investigative the cause for rise in impedance. One may predict such an issue, however, from just six cases there is insufficient data to produce this.
If patients are to be considered for lead extraction, this may be a higher risk procedure.
A suggested management flow diagram is demonstrated on the next slide.
8
Article Reference

## Slide 9
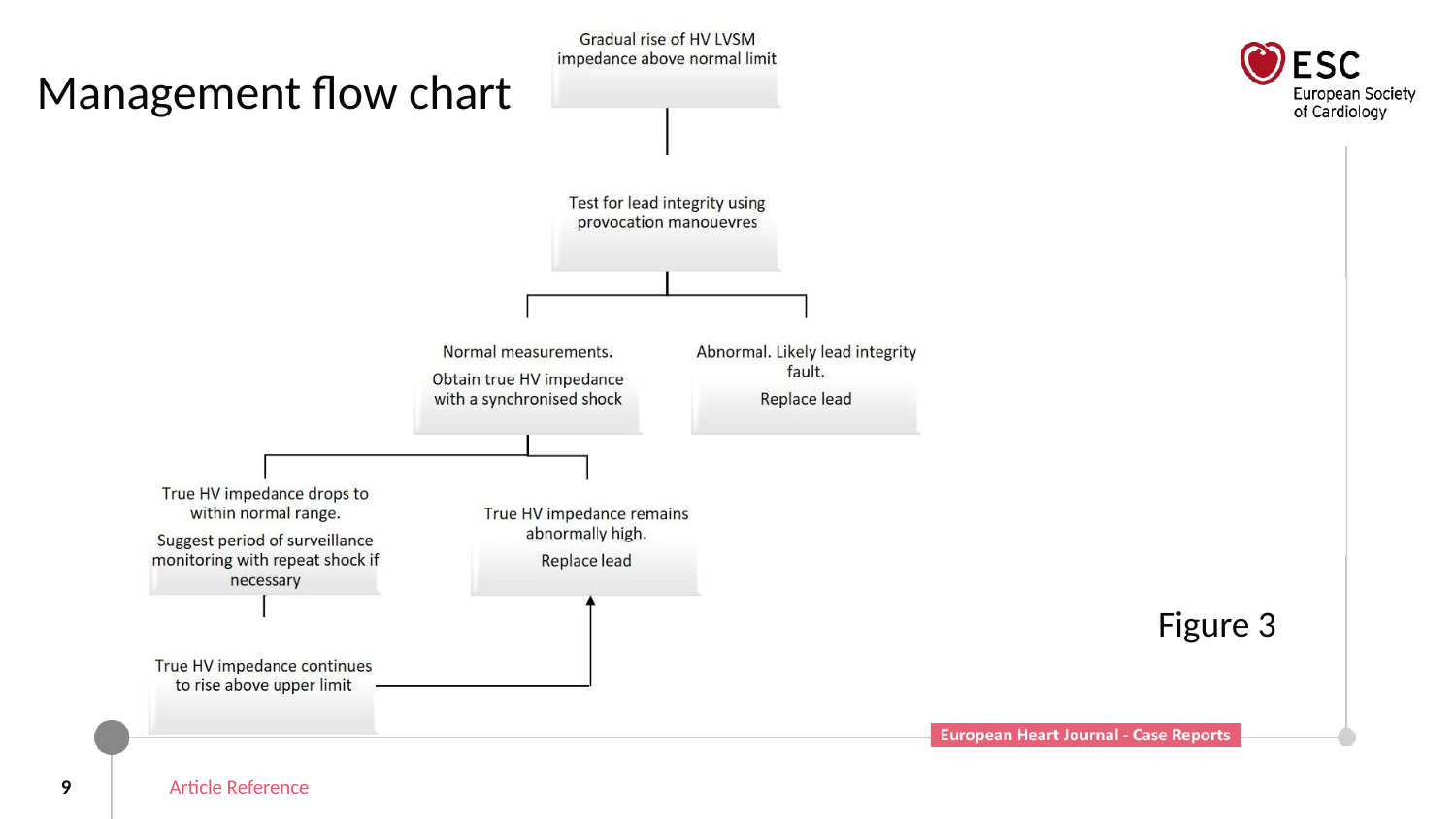

# Management flow chart
Figure 3
9
Article Reference

## Slide 10
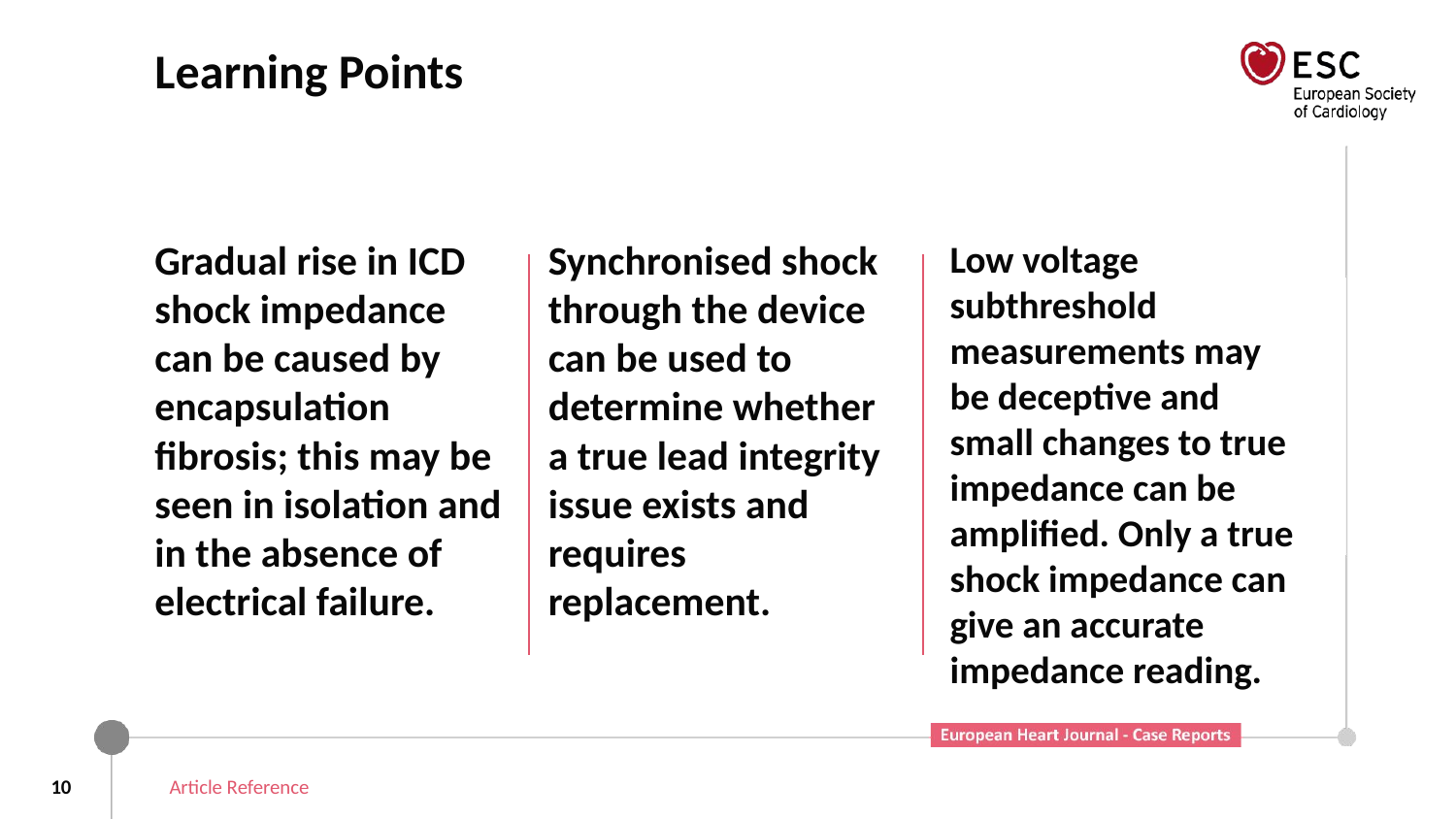

# Learning Points
Gradual rise in ICD shock impedance can be caused by encapsulation fibrosis; this may be seen in isolation and in the absence of electrical failure.
Synchronised shock through the device can be used to determine whether a true lead integrity issue exists and requires replacement.
Low voltage subthreshold measurements may be deceptive and small changes to true impedance can be amplified. Only a true shock impedance can give an accurate impedance reading.
10
Article Reference

## Slide 11
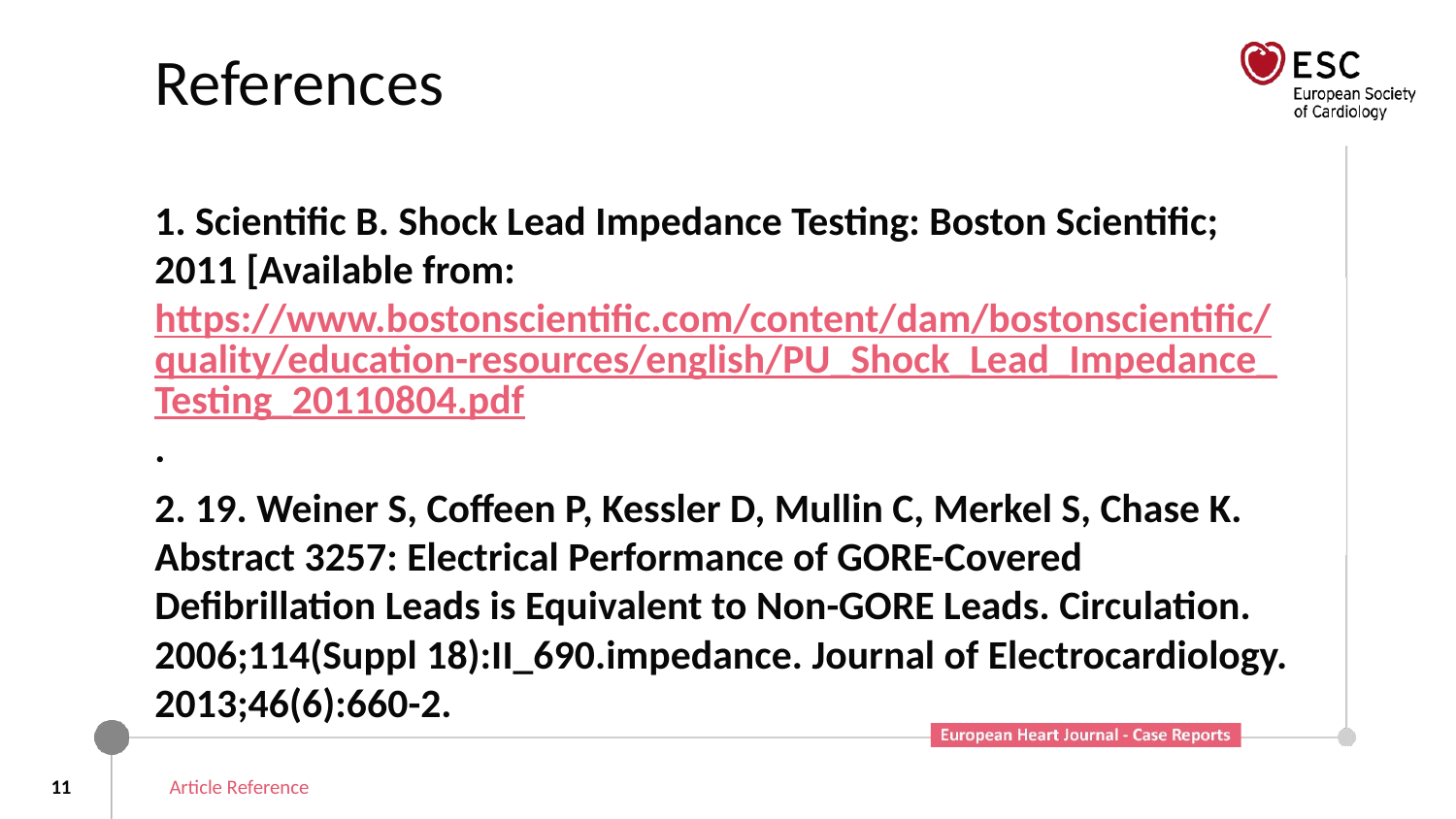

# References
1. Scientific B. Shock Lead Impedance Testing: Boston Scientific; 2011 [Available from: https://www.bostonscientific.com/content/dam/bostonscientific/quality/education-resources/english/PU_Shock_Lead_Impedance_Testing_20110804.pdf.
2. 19. Weiner S, Coffeen P, Kessler D, Mullin C, Merkel S, Chase K. Abstract 3257: Electrical Performance of GORE-Covered Defibrillation Leads is Equivalent to Non-GORE Leads. Circulation. 2006;114(Suppl 18):II_690.impedance. Journal of Electrocardiology. 2013;46(6):660-2.
11
Article Reference
